# Repurposing of Drugs Targeting YAP-TEAD Functions

**DOI:** 10.3390/cancers10090329

**Published:** 2018-09-14

**Authors:** Gian Marco Elisi, Matteo Santucci, Domenico D’Arca, Angela Lauriola, Gaetano Marverti, Lorena Losi, Laura Scalvini, Maria Laura Bolognesi, Marco Mor, Maria Paola Costi

**Affiliations:** 1 Department of Life Sciences, University of Modena and Reggio Emilia, 41125 Modena, Italy; 178871@studenti.unimore.it (G.M.E.); matteo.santucci@unimore.it (M.S.); angela.lauriola@unimore.it (A.L.); 2 Department of Biomedical, Metabolic and Neural Sciences, University of Modena and Reggio Emilia, 41125 Modena, Italy; domenico.darca@unimore.it (D.D.); gaetano.marverti@unimore.it (G.M.); 3 Department of Life Sciences, University of Modena and Reggio Emilia, Unit of Pathology, 41124 Modena, Italy; lorena.losi@unimore.it; 4 Dipartimento di Scienze degli Alimenti e del Farmaco, Università degli Studi di Parma, Parco Area delle Scienze 27/A, 43124 Parma, Italy; laura.scalvini@unipr.it (L.S.); marco.mor@unipr.it (M.M.); 5 Department of Pharmacy and Biotechnology, Alma Mater Studiorum, University of Bologna, 40126 Bologna, Italy; marialaura.bolognesi@unibo.it

**Keywords:** Hippo pathway, YAP-TEAD disruption, drug repurposing, cell signaling, protein-protein interactions

## Abstract

Drug repurposing is a fast and consolidated approach for the research of new active compounds bypassing the long streamline of the drug discovery process. Several drugs in clinical practice have been reported for modulating the major Hippo pathway’s terminal effectors, namely YAP (Yes1-associated protein), TAZ (transcriptional co-activator with PDZ-binding motif) and TEAD (transcriptional enhanced associate domains), which are directly involved in the regulation of cell growth and tissue homeostasis. Since this pathway is known to have many cross-talking phenomena with cell signaling pathways, many efforts have been made to understand its importance in oncology. Moreover, this could be relevant to obtain new molecular tools and potential therapeutic assets. In this review, we discuss the main mechanisms of action of the best-known compounds, clinically approved or investigational drugs, able to cross-talk and modulate the Hippo pathway, as an attractive strategy for the discovery of new potential lead compounds.

## 1. Introduction

**Figure 1:**
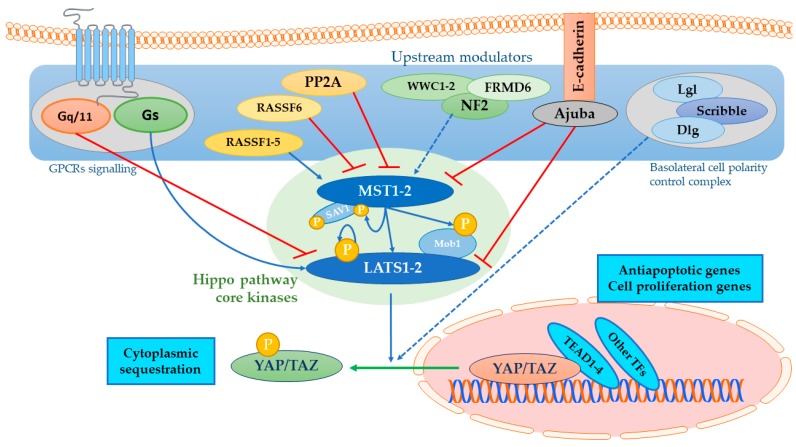
Hippo pathway upstream modulation. Upstream modulators in the blue field regulate Hippo core kinases in the green field, namely MST (mammalian STE20-like protein kinase) 1-2 and LATS (large tumor suppressor) 1-2 [1]. Once phosphorylated, these kinases inhibit YAP (Yes1-associated protein)/TAZ (transcriptional co-activator with PDZ-binding motif) nuclear translocation through serine-phosphorylation, ensuring cytoplasmic compartmentalization and promoting degradation mechanisms. The Hippo pathway induces an onco-protective signal by impairing antiapoptotic and cell proliferation-related genes transcription by the final effectors. G-protein coupled receptors with Gs promote LATS1-2 activation, while other coupling-type mechanisms promote actin cytoskeleton dynamics that impair Hippo kinases activity [12]. The Ras-association domain family (RASSF) proteins generally promote Hippo kinases phosphorylation and activation, while phosphatase promotes the opposite. Other upstream modulators such as FRMD6/WWC1-2/NF2 complex activate Hippo kinases in response to cytomechanical response. Ajuba inactivates MST1-2 and LATS1-2 when it is up-regulated [13,14], while apicobasal cell polarity control complexes, such as the Scribble complex, have been proved to display a tumor-suppressive role [15].

The Hippo pathway attracted increasing interest in the past decade for its role in the regulation of cell growth, differentiation, organ size, and tissue homeostasis [1]. The Hippo pathway basically consists of a core kinase cascade containing the Ste-20 family of protein kinase MST1-2, the scaffolding protein Salvador, and large tumor suppressor kinase LATS1-2 to inhibit the transcriptional co-activators YAP (Yes1-associated protein) and TAZ (transcriptional co-activator with PDZ-binding motif). YAP and TAZ are the major effectors of the Hippo signaling pathway. They function as transcription factors along with TEAD (transcriptional enhanced associate domain) in the nucleus, which increases expressions of such target gene as *CTGF* (connective tissue growth factor), *CYR61* (cysteine-rich angiogenic inducer 61), *AXL* (AXL receptor tyrosine kinase), and *BIRC5* (baculoviral inhibitor of apoptosis repeat-containing 5 or *survivin*) [2]. Recent studies have demonstrated that a wide range of upstream regulators of the Hippo pathway are critical for translating cellular signaling into transcriptional responses (Figure 1). Overgrowth phenotypes upon deregulation of the Hippo pathway have prompted investigation into its role in cancer. Some cancer types have shown deregulation of the Hippo proteins [3], with YAP overexpression particularly associated with solid tumors [4] and with epithelial to mesenchymal transition (EMT) [5]. YAP, TAZ, and TEAD represent the most relevant functional proteins in the signaling system and their role as drug targets have been validated [6]. Owing to its relevance, several drug discovery programs aimed to identify new small molecules that can target the YAP/TAZ/TEAD network, effectively impairing the TEAD function [7,8]. However, only recently, some systematic approaches of drug discovery have led to a few leads that will be further developed. Moreover, a different approach based on the repurposing of existing drugs acting on a pathway connected to YAP/TAZ/TEAD protein network can be considered potentially relevant as inducing a downregulation of the system, therefore acting as long distant effectors.

Repurposing refers to finding new uses for clinically approved drugs (or investigational drugs), different from that for which they were originally registered [9]. It is a particularly attractive strategy in terms of a risk-versus-reward trade-off since critical information related to drug-likeness and pharmacokinetic features, dosing, safety, tolerability, formulation, and manufacturing, are already available. Clearly, this can circumvent the long, risky, and expensive discovery and early clinical stages and streamline the entire drug discovery process [9].

Drugs in clinical practice have been reported for targeting the Hippo pathway, through both cross-talking with other signaling networks and directed to YAP-TEAD interaction inhibition [10,11]. These compounds have been assessed in experimental works to modulate Hippo pathway core proteins levels in both phosphorylated and unphosphorylated forms. Given the promising results, there is no doubt that this research area is going to be more exploited by the biomedical research community to obtain prominent lead compounds. Basing on the above knowledge, the drugs and compounds that have been proposed for re-purposing aims may act at different levels within the YAP-TEAD cascade. In the present review, we examine the best-known compounds and drugs that have been shown to modulate the Hippo pathway and discuss the main mechanisms of actions. The compounds are presented on the basis of the main modulated pathway.

## 2. Drugs Targeting Upstream YAP Modulation

**Figure 2:**
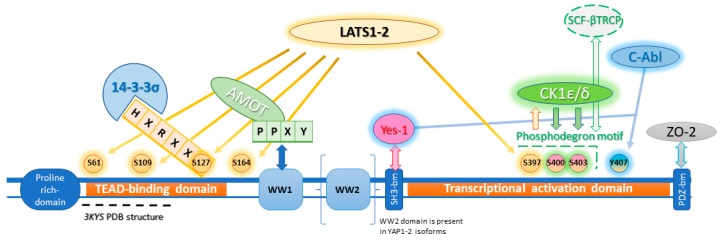
YAP principal interactors and phosphorylation sites. YAP phosphorylation main site is on S127 with consequent 14-3-3σ proteins recruitment, YAP cytoplasmic sequestration and phosphorylation site shielding from phosphatases. LATS1-2 also promotes phosphorylation on other sites (yellow glow), namely S397 phosphorylation which in turn promotes S400 and S403 phosphorylations (green glow) by Casein Kinase 1 family, isoforms δ and ε (CK1δ/ε), overall forming the phosphodegron site responsible for ubiquitination system recruitment [17]. Y407 is phosphorylated by C-Abl and Yes-1 (blue glow) prompting opposite effect according to the involved tyrosine kinase. While C-Abl has onco-protective relevance, promoting binding with p73, Yes-1 promotes nuclear translocation through cross-talking with Wnt/β-catenin pathway (paragraph 2.3.3). The first WW domain (tryptophan tryptophan domain) is responsible for N-terminal AMOT *PPXY* motif binding, while the second one is only conserved in YAP1-2 isoforms. WW1 domain also permits LATS1-2 binding, but it is unclear if this binding is needed for S127 phosphorylation [18]. The terminal motif binds PDZ domain, anchoring YAP to tight junction proteins, such as Zonula Occludens-1/2 (ZO-1/2) [19]. Sequence consensus boxes of the interactors are colored according to the protein to which it belongs [1,20,21].

**Figure 3:**
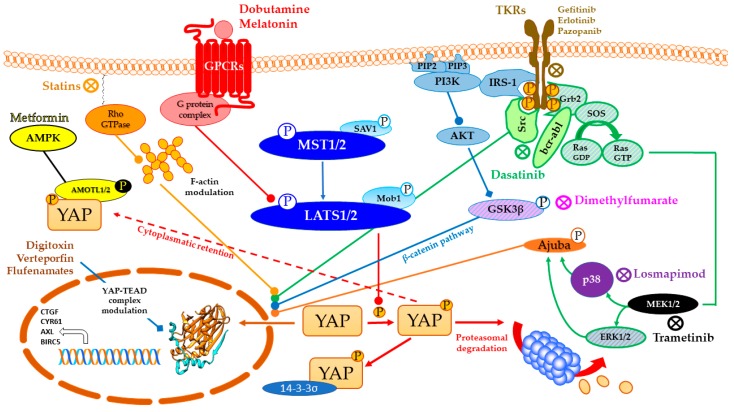
Repurposing of drugs on YAP (Yes-1 associated protein)-TEAD (transcriptional enhanced associate domain) system through cross-talking pathways. Hippo pathway core kinases (in blue) and final effector YAP are modulated by approved drugs through different cross-talking pathways. Dobutamine binds β_1_-adrenergic receptor and promotes LATS (large tumor suppressor) 1/2 phosphorylation through PKA (protein kinase A) signaling, while melatonin is believed to modulate YAP through pleiotropic mechanisms (GPCRs (G-protein coupled receptors) signaling in red). Statins, as HMG-CoA (3-hydroxy-3-methylglutaryl CoA) reductase inhibitors, impair Rho signaling in orange and modulate actin cytoskeleton, impairing LATS1/2 activation altogether. Tyrosine kinase inhibitors target growth factors signaling in cerulean (PI3K-AKT pathway), green (MAPK (mitogen-activated protein kinase) pathway) and black and purple (MAPK pathway final effectors) according to the proper interested drug. Receptor autophosphorylation inhibitors, such as Gefitinib, Erlotinib, and Pazopanib, hit an ATP-binding site. Instead, Dasatinib is a Src/Bcr-abl dual inhibitor, while Losmapimod and Trametinib target downstream components of MAPK pathway. Dimethylfumarate inhibits GSK3β phosphorylation, preventing APC β-catenin destruction complex formation and undermining oncogenic β-catenin pathway in the process. Metformin, through AMPK phosphorylation and consequent half-time AMOT (Angiomotin) prolongment, promotes phosphorylation-independent YAP cytoplasmic sequestration by AMOT in yellow. Digitoxin, Verteporfin and Flufenamic acid and derivatives modulate YAP/TEAD interaction, as better discussed in paragraphs 3 and 4.

YAP phosphorylation preludes to cytoplasmatic sequestration and inactivation by 14-3-3σ proteins and/or AMOT (Angiomotin) and AMOTL1-2 (Angiomotin-like proteins 1-2) [16] or degradation by SCF (Skp, Cullin, F-box complex) β-TrCP (β-transducin repeat-containing proteins) E3 ubiquitin ligase complex, and in this case with a concurrent Wnt pathway suppression [17] (Figure 2 and paragraph 2.4). The following compounds are believed to promote an onco-protective signal mediated by YAP translocation impairment (Figure 3 and Table 1).

### 2.1. GPCRs as Targets to Regulate the Hippo Pathway

GPCRs (G-protein coupled receptors) that act through Gα12/13, Gαq/11 or Gαi stimulate YAP, while Gas coupled receptors have the opposite effect.

Extracellular ligands such as serum-contained lysophosphatidic acid (LPA) and sphingosine-1-phosphate (S1P), that interact with Gα12/13-coupled receptors and thus regulate YAP and TAZ activity through Rho GTPases (and their relative kinase ROCK, Rho-associated protein kinase), lead to actin regulation, which in turn implies LATS1-2 inactivation and subsequent YAP/TAZ activation [12]. Another ligand probably involved in GPCR modulation is thrombin and its expression is increased during blood clotting, a process directly associated with wounds, and may promote wound healing by activating YAP/TAZ through activation of the protease-activated receptor 1 (PAR1) [39]. Somatic mutations interesting *GNAQ* or *GNA11* genes have been correlated to higher YAP activation as a molecular basis of uveal melanoma [40].

Dobutamine, a sympathomimetic amine used as an inotropic agent in heart failure, trough Gs coupling, promotes YAP phosphorylation on S127 site through PKA signaling and so YAP cytoplasmatic retention, confirming in vitro YAP-TEAD transcriptional activity suppression likely involved in F-actin regulation through Rho GTPases, as well as other GPCRs [25].

An interesting hypothesis sheds light on a conjectured pleiotropic mechanism involving a cross-talk between melatonin signaling and the Hippo signaling pathway, possibly foreshadowing implications for cancer therapy. The onco-protective role can be motivated by p38 triggering via MT1/2 binding and possibly by RORE (Retinoic Acid-Related Orphan Receptor Response Element) transcription on retinoic acid response. Melatonin is also proved Reference [41] to counteract estrogen response by impairing calmodulin complex and counterbalance RHOa/ROCK signaling through PKA (protein kinase A) [33]. Melatonin has also been proved to reduce bleomycin (BLM)-induced experimental lung fibrosis in mice by inhibiting YAP nuclear translocation [34].

GPCRs can co-act with IRS-1 (Insulin receptor substrate 1) signaling pathway to up-regulate YAP nuclear translocation. This pathway promotes YAP dephosphorylation, in PDAC (pancreatic ductal adenocarcinoma) cells through PI3K (phosphatidylinositol-4,5-bisphosphate 3-kinase) activation and its downstream effectors, mTOR (mammalian target of rapamycin) signaling pathway, and GSK3β [42] by impairing the PI3K-AKT pathway such as in the case of statins and kinases inhibitors.

### 2.2. HMG-CoA Inhibitors

Statins such as HMG-CoA (3-hydroxy-3-methylglutaryl CoA) are reductase competitive inhibitors that are used to treat hypercholesterolemia by blocking the mevalonate pathway and lowering cholesterol. As a side effect, as well as in myopathy, mevalonate lower levels reduce geranylgeranyl-pyrophosphate, required for Rho GTPase prenylation. Consequent and synergic Rho inhibition demonstrates involvement of an unknown kinase, rather than LATS1-2, in mediating a non-canonical Hippo signaling. These implications are not probably going to be exploited for therapeutic needs because doses required in in vitro studies were higher than the clinical ones [29].

As a further confirmation, through bioinformatical methods, the Mutations and Drugs Portal (MDP) was queried, relying on genomic data extracted from the Cancer Cell line Encyclopedia and the NCI60 DTP projects to obtain a pharmacogenomic association constituted by statins and Dasatinib as an efficient co-targeting strategy [22].

### 2.3. Kinases Inhibitors

MAPK (Mitogen-activated protein kinase) pathway is a mitogenic signaling cascade naturally opposing the onco-protective Hippo kinases by promoting cell growth and proliferation. This pathway is deregulated in 30% of all cancers. Mutations in *KRAS* and *BRAF*, two genes implied in this pathway, have been identified as responsible for 36% and 9–11% of colorectal cancers, respectively [43].

#### 2.3.1. EGFR ATP-Ase Tyrosine Kinase Inhibitors (EGFR TKIs)

Loss of YAP by siRNA (small interfering RNA) or YAP inhibition through Verteporfin treatment, has been reported to enhance Erlotinib cytotoxicity to NSCLC (non-small-cell lung carcinoma) cells H1975 [27]. Autocrine loop with ERBB2 (EGFR family of tyrosine kinase receptors) induced by YAP to bypass EGFR (epidermal growth factor receptor) inactivation regulates ovarian cancer initiation and progression through Hippo kinases inhibition [44]. Different EGFR mutations are distinctly expressed in lung adenocarcinoma cells in which EGFR TKIs frequently occur [23]. Even in this case reducing YAP expression by shRNA (short hairpin RNA) or YAP inhibitors leads to augmented sensitivity. Clinical studies have shown TKIs (tyrosine kinase inhibitors) inefficacy in dealing with resistance mechanisms [45]; even though Dasatinib stood out in terms of cytotoxicity, results have been deemed insufficient to move forward, underlining the importance of YAP inhibition. YAP inhibition was obtained with statin, effectively prolonging survival among lung cancer patients, corroborating the effectiveness of co-targeting EGFR and YAP as a therapeutic strategy through TKI-resistance development delay [23]. Moreover, YAP has been proved to up-regulate EGFR signaling, prompting resistance mechanisms in esophageal cancer [28]. Furthermore, TAZ is over-expressed in NSCLC cells carrying EGFR-T790M mutation, leading to Gefitinib-resistant phenotype and EMT [31].

#### 2.3.2. RAF/MEK Inhibitors

YAP knockout enhances RAF (Rapidly Accelerated Fibrosarcoma) and MEK (MAPK/ERK Kinase) inhibitors efficacy in many RAS/BRAF-mutant tumor types, controlling the threshold of apoptosis, through antiapoptotic genes upregulation [38]. YAP protein level and transcriptional activity of the Hippo pathway in NSCLC cell lines have been reported to be decreased by MEK1-2 inhibitor trametinib [38]. It has been suggested by previous studies that ERK1-2 (extracellular signal–regulated kinases) inhibition participates in reducing the YAP protein level, which in turn down-regulates expression of the downstream genes of the Hippo pathway to suppress migration and invasion of NSCLC cells [46].

#### 2.3.3. Bcr-Abl and the Src Kinase Family TKi

Bcr-Abl first generation drug Imatinib was proved to increase YAP suppression in chronic myeloid leukemia (CML) cells promoted by Verteporfin, involving PI3K-Akt and MAPK pathways as previously seen [47]. Conversely, Dasatinib also inhibits Src family kinases and decrease YAP/TAZ nuclear translocation, through changes of actin dynamics. Suppression of proliferation of β-catenin-active cell lines in in vitro depends on the inhibition of YES1 and the resulting inactivation of the YAP–β-catenin–TBX5 complex, interrupting the process the nuclear localization of YAP, by also regulating the actin dynamics and through YES1-mediated YAP reduction. In fact, YAP nuclear localization and stabilization is performed by Dasatinib by reducing Yes1-promoted and activating YAP Y357 phosphorylation (position in YAP1 iso3 (YAP1-1β) corresponding to Y407 in YAP1 iso1 (YAP1-2γ) [20,21]) or by increasing YAP S127 phosphorylation via LATS1-2 [24].

#### 2.3.4. Other Kinases Inhibitors

When Ajuba, adapter or scaffold protein which participates in the assembly of numerous protein complexes and involved in several cellular processes, is up-regulated, a consequent Hippo kinasic activity suppression occurs through LATS1-2 binding, preventing its phosphorylation on the YAP S127 phosphorylation site [48]. This complex is involved in γ-tubulin recruitment in centrosomes and in mitotic spindle organization [49]. Terminal effectors of MAPK signaling evoke Ajuba, abrogating Hippo kinasic onco-protective activity [13,14], explaining Losmapimod activity, by blocking p38 mitogen-activated protein kinases [32]. It should be noted that p38 MAPKs can either promote or inhibit cell growth depending on the type of the stimulus or the involved cell type [50].

Pazopanib, a c-KIT (mast/stem cell growth factor receptor), FGFR (fibroblast growth factor receptor), PDGFR (platelet-derived growth factor receptor), and VEGFR (vascular endothelial growth factor receptor) multi-kinase inhibitor induced the proteasome-dependent degradation of YAP and TAZ, which was not prominent in the cells treated with Dasatinib and Fluvastatin, besides interrupting YAP nuclear localization altogether [30,37].

### 2.4. GSK3β Phosphorylation Inhibition

Wnt pathway stimulation via Frizzled receptor activation causes Dsh (Dishevelled) inhibiting GSK3β phosphorylation and activation [51,52]. Without this activation, GSK3β no longer promotes β-catenin phosphorylation, with consequent nuclear accumulation and transcriptional complex formation with Lef/Tcf (T-cell factor/lymphoid enhancer-binding factor), and APC (adenomatous polyposis coli) β-catenin destruction complex at cytoplasmic level with Axin can not either be formed. Wnt and β-catenin pathways have a demonstrated responsibility in the colorectal cancer pathogenesis. β-catenin activity study in 85 cancer cell lines found that tumorigenic mechanism involves YAP through the above-mentioned APC complex. YAP phosphorylation through tyrosine kinase YES1, and so in another phosphorylation site rather than the main one, implies involvement of this complex in *BCL2L1* (Bcl-2-like 1) and *BIRC5* transcription [53]. In a similar way of Wnt signaling, dimethylfumarate (DMF) can hinder GSK3β phosphorylation by impairing PI3K/AKT pathway, thus reducing nuclear localization of YAP, but also abrogating TGFβ/Akt1-mediated inhibitory phosphorylation of GSK3β, with consequent proteasomal degradation of YAP. Studies conducted in human Hek293 and in mouse NIH3T3 cells have demonstrated that after YAP phosphorylation at S381 by LATS1-2 (position in YAP1 iso2 (YAP1-2α) corresponding to S397 in YAP1 iso1 (YAP1-2γ) [20,21]), another phosphorylation, possibly performed by casein kinase 1 (CK1δ/ε) that triggers degradation via E3-ubiquitin SCFβ-TRCP, leading to YAP polyubiquitination and degradation [17] (Figure 2). YAP levels diminution could also explain the anti-fibrotic outcomes of DMF, since animal models have demonstrated a role in reversing the pro-fibrotic phenotype of systemic sclerosis dermal fibroblasts. Cytoskeletal changes and YAP nuclear translocation impairment were evaluated replicating healthy and fibrotic tissues stiffness trough polyacrylamide gels with different modulus [26]. Previously, DMF was used to treat autoimmune diseases, like multiple sclerosis and psoriasis, probably involving covalent modifications through Michael addition by cysteine residues, namely Nrf2 (Nuclear factor erythroid-derived 2-like 2) with a consequent anti-oxidant response [54].

### 2.5. AMOT Stabilization by Metformin

Another cytoplasmic compartmentalization is acquired through inhibition by AMOT-mediated tight junction localization mediated by the phosphorylation-independent mechanism.

Metformin has been implied to be involved in cross-talking with Hippo pathway, inhibiting YAP nuclear translocation. Furthermore, energy stress caused by glucose deprivation induces AMOTL1 phosphorylation at S793 by 5′AMP-activated protein kinase (AMPK), which is activated by the growth of AMP/ATP rate caused by mitochondrial respiratory-chain complex 1 block [55,56]. The notion of augmented AMOTL1 protein stability derives from studies indicating a phosphorylation-deficient S793A mutant that showed a shorter half-life that were obtained through AMOTL1 knock-out mediated by shRNA, followed by shRNA-resistant wild-type AMOTL1 or AMOTL1 S793A mutant over-expression [35].

Interestingly, loss of YAP/TAZ by siRNA also demonstrated a similar effect of Metformin towards insulin axis, inhibiting cell growth and implying a novel function of YAP in an insulin resistance IRS1/2-mediated endometrial cancer; Verteporfin obtained similar results as comparison [36].

## 3. YAP/TEAD Interaction Modulation through Direct Ligands Design

Computational techniques, including virtual screening and docking, are commonly used to screen large databases of molecules, allowing the rapid discovery of potential lead compounds. In the case of drug repurposing campaigns, the identification of already-known or marketed compounds with novel target activity can be afforded by these approaches. When the target structure is known, as in the case of TEAD, docking and structure-based studies allow the identification of molecules characterized by complementary shape and electrostatic distribution with their binding sites, and to estimate the interaction energy of ligand-target complexes. The selection of hit compounds, which are forwarded to biological assays, is based on the application of scoring functions that provide an approximation of the binding free-energy, considering the interaction energy and empirical functions that account for desolvation and entropic factors [57,58]. In particular, docking of virtual structures has been applied to devise a possible interaction model for Digitoxin with YAP, as detailed below. A virtual screening campaign, aimed at drug repurposing, led to the identification of Flufenamic acid as an inhibitor of TEAD palmitoylation, as discussed in the next chapter. Specifically, structure-based drug identification approaches have been applied to the identification of pharmaceutical agents for directly interfering with YAP/TEAD signaling pathway (Table 2), rather than regulate upstream mediators as before described.

### 3.1. Cardiac Glycosides as Potential YAP Function Modulators

Sudol et al. [59] have proposed an in silico model for Digitoxin binding with a homology model of YAP WW domain extrapolated from WW domain of dystrophin and obtained with comparative modelling software MODELLER, showing a potential inhibiting role with proteins exposing *PPXY* motif, namely LATS1-2 (Figure 2). This hypothesis has not been confirmed yet by any experimental study, as for now there are no in vitro studies testing YAP affinity. However, docking approaches, besides predicting AMOT and LATS interaction with YAP WW domain, estimated that Digitoxin could arrange an extensive network of Van der Waals interactions and hydrogen bonds with a determined set of residues in the proposed hydrophobic binding groove. It should be noted that this set of residues could permit target selectivity discrimination among the other WW domains [59]. Cardiac glycosides so should be able to mimic interactions of YAP physiological inhibitors at level of *WW1* domain on the YAP protein. Targeting WW domain could impair complexes formation, such as with Wbp2, involved in conveying oncogenic signals through YAP/TAZ activity enhancement [69] or be beneficial in tumors where YAP exerts a potential oncosuppressive role, as seen in breast cancer [70]. As a testament, recent findings suggested that digitoxin promotes YAP nuclear sequestration, phospho-YAP (S127) decrement and downstream transcription factors levels increase, in both HK-2 cells [60] and different human lung squamous cell lines [61]. These outcomes respectively elicit regenerative and fibrogenetic mechanisms after acute kidney injury (AKI), with the prospect of prevent transition to chronic kidney disease (CKD) [60], and suppress cancerous progression through ROS accumulation due to scavenger enzyme genes down-regulation [61], thus prompting the use of YAP agonist, as opposed to the previously discussed cases. Therefore, these data are evidence that YAP role in oncogenesis is so intimately related with tissue environment and cell lineage.

Furthermore, Digitoxin and the other cardiac glycosides have been reported as cytotoxic agents, probably not exclusively due to their activity as Na^+^/K^+^ ATPase inhibitors, with consequent membrane fluidity alterations, increasing Ca^2+^ levels. Additionally, through mitochondrial Na^+^/Ca^2+^ pump activity exacerbation and triggering cytoskeleton alterations, that lead to cell cycle dysregulation with possible Mcl-1 (induced myeloid leukemia cell differentiation protein Mcl-1) and Bcl-xl (B-cell lymphoma-extra large) down-regulation, it is a possible a mechanism involving a mitochondrial apoptosis-inducing pathway [71,72]. Other hypotheses involve an ampler signalosome [73,74] leading to a multitude of effects leading to cell death, namely topoisomerase inhibition [75], EGFR/Src/Akt signaling cross-talking [76], Na^+^/K^+^ ATPase, and Src endosomal trafficking and immunogenic cell death [74], p21 up-regulation and JNKs (c-Jun N-terminal kinases) activation [77], and HIF1-α (hypoxia-inducible factor 1-alpha) inhibition [78].

### 3.2. Verteporfin and Derivatives as YAP-TEAD Interaction Disruptors

Liu-Chittenden et al. [62] identified Verteporfin (VP, Visudyne^®^ by Novartis) and two other protoporphyrins, Protoporphyrin IX (PPIX), and Hematoporphyrin (HP) as YAP-TEAD interaction inhibitors. They first set up a luciferase reporter assay through Gal4-TEAD4 fusion protein expression in order to screen Johns Hopkins Drug Library and, leveraging on intrinsic VP fluorescence and through proteolytic patterns comparison, they clarified a selective binding to YAP. Co-immunoprecipitation assays revealed that both VP and PPIX abolish YAP-TEAD complex formation at 10 µM, but the first molecule with greater potency [62]. Since VP is adopted by photodynamic therapy, it was necessary to prove activity in an environment without light as it was reported in a study where levels of YAP, phospho-YAP (S127), CYR61 and CTGF were diminished in uveal melanoma cells [63]. In a subsequent research regarding growth inhibition in human glioma cells in vitro, as well as implying p38 activation with its consequent antiproliferative role [64]. The same study also underlined the possibility of treatment of different tumor cell lines, namely retinoblastoma cells [65], ovarian cancer cells [66], and endometrial cancer cells [79]. VP also downregulates EGFR levels, as these are dependent on YAP-promoted transcriptional activity, effectively lowering chemoresistance to cytotoxic drugs [28].

YAP is conceivably targeted with different modes, since 14-3-3 proteins up-regulation is promoted with consequent YAP cytoplasmic sequestration, suggesting a VP scaffold-like phosphorylation-mimicking interaction concerning the *HXRXXS* motif (Figure 2) [67].

Gibault et al. [80] synthetized molecular simplification derivatives, among which a symmetric divinyldipyrrine stands out regarding its inhibitory activity. Nevertheless, due to vinyl groups, it is unclear whether this result is obtained through extensive proteotoxicity [80].

It has to be noted that VP could also promote oligomerization of high-molecular weight proteins, obstructing proteasomal and autophagic degradation systems on which cancer cells heavily rely to hunt nutrients for cell growth [81]. Formation of high-molecular weight complexes have also been assessed in different types of cancer cell lines as a light-mediated post-lysis effect [82].

## 4. Drugs Directed to the Palmitoylation Pocket of TEAD

**Figure 4:**
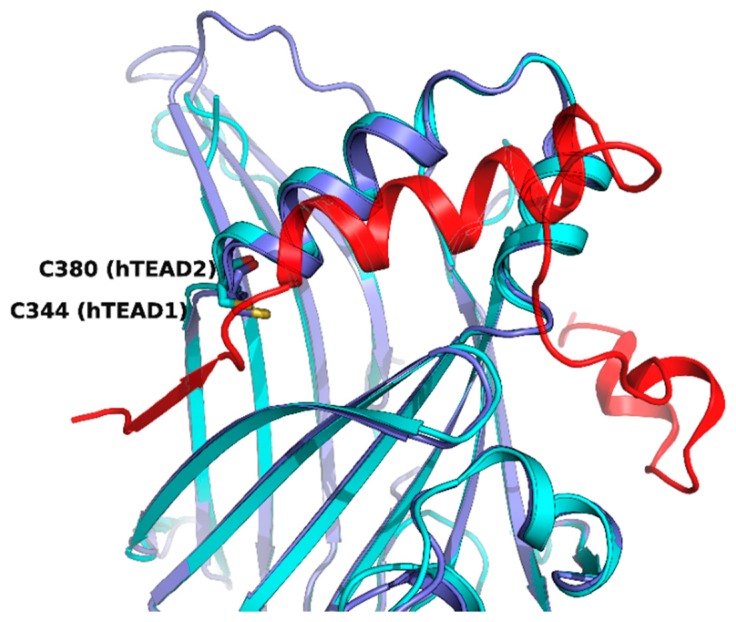
Superposition of hTEAD2 (as part of the PDB structure *5DQ8*, in cyan) and hTEAD1 in complex with YAP2 (in blue and red, respectively, as part of the PDB structure *3KYS*). The β1 strand of YAP is displayed on the left, the α1 helix in the center, and the Ω-loop on the right of the figure. The cysteines which are the sites of palmitoylation for the two TEAD isoforms are evidenced.

While the N-terminal domain of human TEAD1-4 is involved in DNA recognition, the C-terminal regions interact with YAP, and a strong protein-protein interaction is required for the formation and stabilization of the YAP/TEAD complex, which promotes gene transcription. Three motifs of YAP (a β-strand called β1, a α-helix called α1 and a twisted-coil region called Ω-loop) are placed around the surface of TEAD formed by a portion of the two β-sheets, which form a β-sandwich in the core of the YAP-interacting domain, and by a helix-turn-helix motif which connects the two sheets (Figure 4). While the three portions of YAP contribute differently to the strength of YAP/TEAD association, the synergism conferred by the presence of all of them is responsible for the high affinity of the complex [83,84,85].

Therefore, another potentially successful strategy to impair YAP-TEAD interaction could be addressing this TEAD central pocket, directly targeting the final effector in genic expression regulation and contemporarily exploiting a conserved hydrophobic site with good druggability. TEAD has a highly conserved palmitoylation site (C344 in TEAD1, C380 in TEAD2, C371 in TEAD3, C360 in TEAD4) [86]. The residue numbers are reported as in the available crystallographic structures, although in the case of TEAD1, they do not correspond to the Uniprot sequence numeration (i.e., residue C344 in the X-ray structure corresponds to C359) [84]. The palmitoylation site is close to the surface interacting with YAP/Vgll4 (vestigial-like family member 4) β1, so it is plausible to inquire that displacing the palmitoyl-moiety could disrupt protein-protein interface through allosteric communication.

While trying to identify autopalmitoylated proteins and studying PATs (palmitoyl acyl-transferases), Chan et al. [87] also identified TEAD1 and TEAD3 as palmitoylated proteins by synthetizing chemical probes as 2-bromohexadec-15-ynoic acid and cis-2,3-epoxy-4-oxooctadec-17-ynamide. These probes were obtained with the specific purpose to gain analogues of palmitic acid for click chemistry—as done before by the same researchers to study Wnt palmitoylation [88]. To validate chemoproteomical results, the authors performed, with labelled FLAG-coimmunoprecipitated proteins, a Huisgen 1,3-dipolar cycloaddition with biotinylated azide, recognized by streptavidin western blot analysis, which detected all TEADs paralogues as palmitoylated through reversible thioester bond. These findings suggested that such a post-translational modification is evolutionarily conserved, by also individuating the cysteine residue majorly involved in all paralogues. Past X-ray resolved structures were critically re-evaluated linking pocket electronic extra-density to palmitoylate, rather than to other interferents, and compared those with recently obtained (*PDB codes 5EMV/5EMW* with TEAD2/3). The importance of the palmitoylate as a fold stability hub was proved through fluorescence resonance energy transfer (FRET) AlphaScreen assays by using TEAD1 mutant C359S (corresponding to C344 in the crystallographic structures), which showed a weaker binding to YAP respect to wild-type TEAD, but not with Vgll4. They also found that DHHC-family PATs overexpression did not prompt any sizable fluctuation in palmitoylation levels, suggesting a probable autopalmitoylation mechanism [87]. Furthermore, they found Scribble pro-oncogenic mislocalization away from the cell-cell junction as a result of ZDHHC7 (zinc finger DHHC domain-containing protein 7) knock out, as they demonstrated that palmitoylation promoted role of Scribble in YAP activity suppression and cell polarity control [15]. Another team has individuated TEAD-palmitoylation as a process rather slower than expected, observing palmitoylation levels reduction through acylation with Ω-alkynil palmitic acid, but that it strangely does not affect cell localization, since TEAD is not stably associated to nuclear envelope. To inquire into the role of S-palmitoylation, they tested TEAD2 mutants C380A and K357A in HEK293T cells, as they predicted that neighboring lysine amino group could lower cysteine the pKa group, promoting palmitoylation in the process [86].

**Figure 5:**
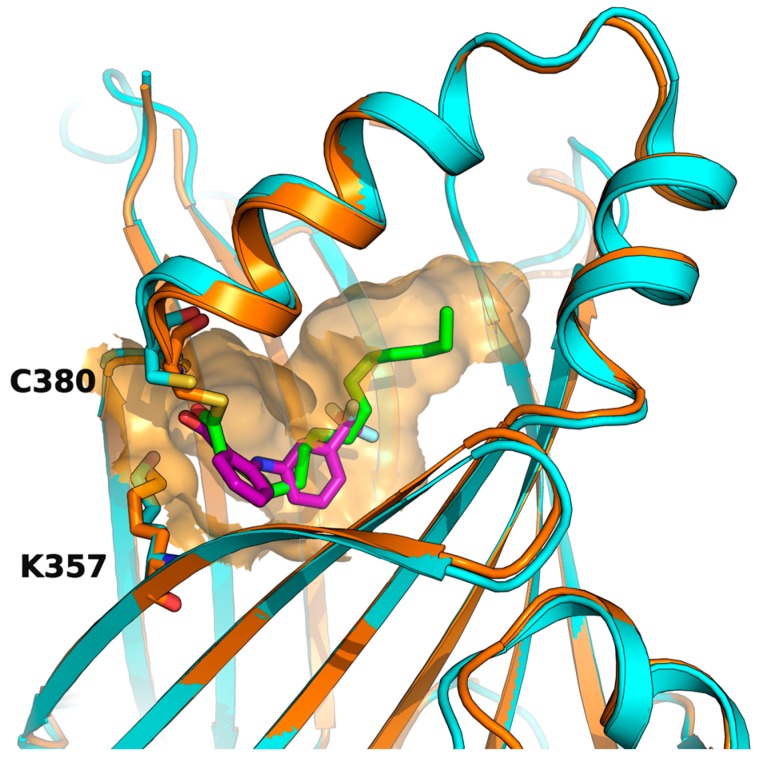
Flufenamic acid (in purple as part of the PDB structure *5DQ8*, in cyan) and palmitate (in green as part of the PDB structure *5EMV*, in orange) share the same binding pocket as shown by superposition of co-crystallized hTEAD2 structures. The surface of the amino acid residues lining the binding pocket of hTEAD2 is represented in orange.

Before the palmitoylation of TEAD was reported, Pobbati et al. [68] had already shown the druggabiliy of this central hydrophobic pocket. In silico screening of a Pharmakon library built from FDA approved entities, followed by differential scanning fluorimetry measuring TEAD4-YBD (YAP-binding domain) Tm change, identified Flufenamic acid as a TEAD-binding drug. Flufenamic acid binding was then confirmed with STD-NMR (saturation-transfer difference-NMR) and with ITC they were able to find Kd = 73 µM. Crystal structure of TEAD2-YBD confirmed Flufenamic acid (*PDB* code *5DQ8*) or bromofenamic acid (*PDB* code *5DQE*) electron density in the palmitoylation pocket. The carboxylate group of the fenamates takes a hydrogen bond with the backbone nitrogen of C380, as well as a salt bridge with K357 (Figure 5). However, the fenamates class did not show any involvement in interaction disruption, even if, at a higher dose, luciferase reporter assay showed TEAD activity reduction. Nowadays, the role of palmitoylation, and of its displacement on the YAP-TEAD interaction, is still controversial. In fact, Mesrouze et al. have recently reported that YAP and TAZ bind in a similar manner to acylated and non-acylated TEAD4 [89].

## 5. Conclusions

Given the results obtained in other fields, there is no doubt that repurposing attractive research areas is going to be more and more exploited in a pragmatic way to obtain prominent lead compounds. While drug repurposing relies on existing drugs with already safety outline, and approved clinical use, it is evident that medicinal chemists still play a crucial role in the context of these efforts. This is because it is extremely unlikely that the repurposed drugs are already optimized for the most favorable interaction with YAP-TEAD network proteins. Thus, it is highly feasible that they will lead to the best future outcomes, through projects aimed at identifying optimized analogs with improved activities, reduced toxicities, and better pharmacokinetic properties tailored on appropriate target product profiles, for a wide range of therapeutic applications, especially in the antitumor field.

## Figures and Tables

**Table 1:** Pharmaceutical agents targeting Hippo signaling through cross-talking pathways and their approved clinical indication.

Active Compound	Common Name	Mechanism of Action	Indication	References
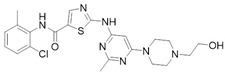	**Dasatinib**	BCR-ABL inhibitor Src family tyrosine kinase inhibitor	Acute Lymphoblastic Leukaemia (ALL) Chronic Myeloid Leukaemia (CML)	[22,23,24]
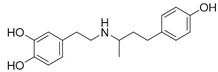	**Dobutamine**	β-1 adrenergic receptor agonist	Cardiac decompensation Coronary artery disease	[25]
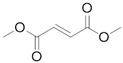	**Dimethylfumarate**	Nrf2 cysteine covalent modification (GSK3β phosphorylation block)	Relapsing remitting multiple sclerosis	[26]
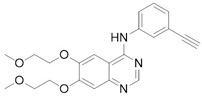	**Erlotinib**	EGFR inhibitor	Non-small-cell lung carcinoma (NSCLC) Metastatic pancreatic cancer	[27,28]
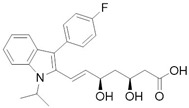	**Fluvastatin**	HMG-CoA reductase	Atherosclerosis Hypercholesterolemia	[29,30]
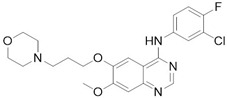	**Gefitinib**	EGFR inhibitor	Metastatic Non-Small Cell Lung Cancer	[23,31]
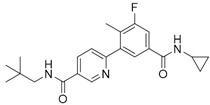	**Losmapimod**	p38 MAPK inhibitor	Investigational	[32]
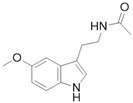	**Melatonin**	MT1/2 receptors agonist	Sleeplessness	[33,34]
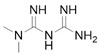	**Metformin**	AMPK activator Mitochondrial complex I inhibitor	Type II diabetes	[35,36]
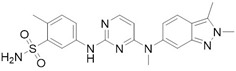	**Pazopanib**	c-KIT, FGF, PDGF, VEGF receptors inhibitor	Advanced Renal Carcinoma Advanced Soft Tissue Carcinoma	[30,37]
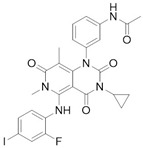	**Trametinib**	MEK1/2 inhibitor	Metastatic melanoma	[38]

**Table 2:** Pharmaceutical agents directly modulating YAP-TEAD interaction.

Active Compound	**Common Name**	**Mechanism of Action**	**Indication**	**References**
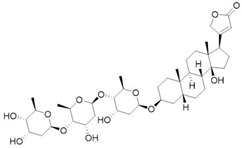	**Digitoxin**	Na^+^/K^+^ ATPase inhibitor	Congestive cardiac insufficiency Heart failure	[59,60,61]
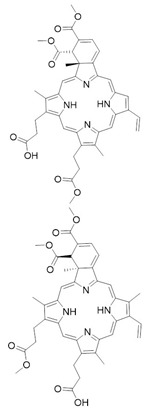	**Verteporfin**	Photodynamic agent	Subfoveal Choroidal Neovascularization	[28,62,63,64,65,66,67]
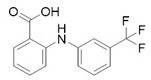	**Flufenamic Acid**	COX-inhibitor	NSAID	[68]
